# Alteration in gut microbiota is associated with immune imbalance in Graves’ disease

**DOI:** 10.3389/fcimb.2024.1349397

**Published:** 2024-03-12

**Authors:** Yalei Liu, Shasha Tang, Yu Feng, Binghua Xue, Chaofei Cheng, Yong Su, Wei Wei, Lijun Zhang, Zhoufeng Huang, Xiaoyang Shi, Yuanyuan Fang, Junpeng Yang, Yun Zhang, Xinru Deng, Limin Wang, Hongyan Ren, Chongjian Wang, Huijuan Yuan

**Affiliations:** ^1^ Department of Endocrinology, Henan Provincial Key Medicine Laboratory of Intestinal Microecology and Diabetes, Henan Provincial People’s Hospital, People’s Hospital of Zhengzhou University, Zhengzhou, Henan, China; ^2^ Stem Cell Research Center, Henan Provincial People’s Hospital, People’s Hospital of Zhengzhou University, Zhengzhou, Henan, China; ^3^ Institution of Hematology, Henan Provincial People’s Hospital, People’s Hospital of Zhengzhou University, Zhengzhou, Henan, China; ^4^ Shanghai Mobio Biomedical Technology Corporation Limited, Shanghai, China; ^5^ Department of Epidemiology and Biostatistics, College of Public Health, Zhengzhou University, Zhengzhou, Henan, China

**Keywords:** Graves’ disease, gut microbiota, B cells, LPS, cytokines

## Abstract

**Background:**

Graves’ disease (GD), characterized by immune aberration, is associated with gut dysbiosis. Despite the growing interest, substantial evidence detailing the precise impact of gut microbiota on GD’s autoimmune processes remains exceedingly rare.

**Objective:**

This study was designed to investigate the influence of gut microbiota on immune dysregulation in GD.

**Methods:**

It encompassed 52 GD patients and 45 healthy controls (HCs), employing flow cytometry and enzyme-linked immunosorbent assay to examine lymphocyte and cytokine profiles, alongside lipopolysaccharide (LPS) levels. Gut microbiota profiles and metabolic features were assessed using 16S rRNA gene sequencing and targeted metabolomics.

**Results:**

Our observations revealed a disturbed B-cell distribution and elevated LPS and pro-inflammatory cytokines in GD patients compared to HCs. Significant differences in gut microbiota composition and a marked deficit in short-chain fatty acid (SCFA)-producing bacteria, including ASV263(*Bacteroides*), ASV1451(*Dialister*), and ASV503(*Coprococcus*), were observed in GD patients. These specific bacteria and SCFAs showed correlations with thyroid autoantibodies, B-cell subsets, and cytokine levels. In vitro studies further showed that LPS notably caused B-cell subsets imbalance, reducing conventional memory B cells while increasing naïve B cells. Additionally, acetate combined with propionate and butyrate showcased immunoregulatory functions, diminishing cytokine production in LPS-stimulated cells.

**Conclusion:**

Overall, our results highlight the role of gut dysbiosis in contributing to immune dysregulation in GD by affecting lymphocyte status and cytokine production.

## 1 Introduction

Graves’ disease (GD), a targeted autoimmune disorder, impacts approximately 2% of women and 0.2% of men globally (McLeod and Cooper, 2012; Smith and Hegedüs, 2016; Taylor et al., 2018), with a higher prevalence among individuals aged 30 to 50 years (Liu H. et al., 2022). Epidemiological studies have revealed that GD manifests at a rate of about 20 to 40 cases per 100,000 individuals annually (Davies et al., 2020). The disease manifests through the diminished self-tolerance to thyroid antigens, particularly the thyroid-stimulating hormone receptor (TSHR), accompanied by lymphocytic infiltration within the thyroid tissue (Weetman, 2001). The antibody targeting TSHR (TRAb) function predominantly as an agonist, precipitating unchecked hormone secretion and leading to the prevalent symptom in GD patients: hyperthyroidism (Davies et al., 2020). This hyperthyroid condition is linked to heightened risks for several health issues, such as atrial fibrillation, pulmonary embolism, and preeclampsia (Brandt et al., 2011). Despite these insights, the exact mechanisms underlying GD’s onset remain enigmatic.

In GD, the infiltration of lymphocytic is predominantly composed of CD4^+^ T cells, CD8^+^ T cells, and CD19^+^ B cells (Kristensen B., 2016). While T cells contribute to autoimmunity through cytokine release and by providing activation signals to B cells (Bonasia et al., 2021), their exact impact hinges on their specific subsets. CD4^+^ helper T (Th) cells can be subdivided into functionally dis tinct subsets, including Th1, Th2 and Th17 cells, while CD8^+^ cytotoxic T (Tc) cells can be classified into Tc1, Tc2 and Tc17 cells. Although T-cell autoreactivity is evident, B cells seemingly spearhead the synthesis of thyroid autoantibodies, encompassing TRAb, thyroid peroxidase antibody (TPOAb), and thyroglobulin antibody (TgAb). IgD is mainly expressed on naïve B cells and functions to inhibit response, while CD27 promotes B cells terminal differentiation (Han et al., 2016; Nechvatalova et al., 2018). Based on CD27 and IgD expression, B cells can be subdivided into four classical subsets: naïve (CD27^-^IgD^+^), pre-switched memory (CD27^+^IgD^+^), double-negative memory (CD27^-^IgD^-^) and conventional memory (CD27^+^IgD^-^) subsets. An atypical distribution of B-cell subsets, combined with hindered immune checkpoint molecule expression, is postulated to ignite humoral immune activation (Smith and Clatworthy, 2010; Jin et al., 2020). This cascade amplifies autoantibody production, inflicting substantial harm to the host. Our previous study has proved abnormal distribution of B-cell subsets occur in the presence of GD (Liu Y. et al., 2022), yet the exact mechanisms initiating this immune response remain to be fully understood.

Recently, the role of gut microbiota in modulating immunological homeostasis has garnered significant interest. A mounting body of evidence indicates alterations in the gut microbiota composition among patients with autoimmune disorders, encompassing systemic lupus erythematosus (Guo et al., 2020), rheumatoid arthritis (Xu et al., 2020), inflammatory bowel disease (Palm et al., 2014) and type 1 diabetes mellitus (Biassoni et al., 2020). Recent investigations have elucidated marked gut microbiota dysbiosis in GD patients, characterized by reduced diversity and skewed microbial profiles (Su et al., 2020; Jiang et al., 2021). Nonetheless, certain studies yield conflicting data concerning the genera levels (Zhao et al., 2022; Deng et al., 2023). Notably, a majority of these investigations rely on clustering sequences based on operational taxonomic units (OTUs) — an approach that has faced scrutiny (Edgar, 2018). Given the nebulous understanding of the precise interplay between autoimmune activation and gut microbiota in GD, there is an urgent need for more accurate methodologies, such as amplicon sequence variant (ASV) analysis, to investigate the impact of gut microbiota on GD autoimmunity. Thus, this study aims to examine the influence of gut microbiota on immune abnormalities in GD using the ASV analysis method.

In the study, we initially contrasted the immune profiles, gut microbiota, and short-chain fatty acids (SCFAs) between GD patients and healthy controls (HCs). We then delineated the interrelationships among pivotal ASVs, SCFAs, immune-inflammatory indices, and clinical phenotypes. Subsequently, we investigate the effect of SCFAs and lipopolysaccharides (LPS) on immune status in an *in vitro* study. The results of the study may provide novel insights into the autoimmune pathogenesis of GD.

## 2 Materials and methods

### 2.1 Study population and sample collection

A total of 52 GD patients and 45HCs were recruited from Henan Provincial People’s Hospital from March 2020 to March 2021. Demographic details, clinical data and dietary habits of the participants were collected from hospital electronic medical records and questionnaires. Comprehensive diagnostic criteria, along with inclusion and exclusion criteria for the participants, are elucidated in the **Supplementary Methods**.

The study was approved by the Ethics Committee of Henan Provincial People’s Hospital [NO. 2019 (66)]. All procedures were executed in compliance with the Declaration of Helsinki, and written informed consents were obtained from all study participants.

All participants underwent an overnight fast of no less than 8 hours. Both fecal and serum specimens from all participants were harvested and preserved at −80°C pending DNA extraction or cytokine assessment. Additionally, fresh whole blood samples from 33 GD patients and 32 HCs were collected and subjected to immediate flow cytometry analysis.

### 2.2 Laboratory testing

Thyroid function tests and thyroid autoantibody assessments were conducted at the clinical laboratory of Henan Provincial People’s Hospital using chemiluminescent immunoassays techniques. Measurements of serum levels of TSH, free triiodothyronine (FT3), free tetraiodothyronine (FT4) and TRAb were performed with the Cobas e602 analyzer (Roche Diagnostics, Basel, Switzerland). This analyzer utilizes ruthenium-complex-labeled antibodies, including anti-TSH, anti-T3, anti-T4 antibodies, and the human TSHR monoclonal antibody M22. Additionally, serum levels of TgAb and TPOAb were measured using the UniCel DxI 800 analyzer (Beckman Coulter, Brea, USA). These assays were executed following the manufacturer’s instructions provided in the package inserts. The established reference values for normalcy are delineated as: FT3 (3.1-6.8 pmol/L), FT4 (12.0-22.0 pmol/L), TSH (0.27-4.2 μIU/mL), TgAb (0-4 IU/mL), TPOAb (0-9 IU/mL), and TRAb (< 1.75 IU/L).

### 2.3 DNA extractions and 16S rRNA gene amplification sequencing

Genomic DNA was extracted from fecal samples using the E.Z.N.A. ^®^Stool DNA Kit (Omega Biotek, Inc., USA). The harvested DNA served as the template for PCR amplification targeting the V3-V4 region of the 16S rRNA genes. Utilized forward primer (341F) had the sequence 5’-CCTACGGGNGGCWGCAG-3’, and the reverse primer (805R) was 5’-GACTACHVGGGTATCTAATCC-3’. Amplification was conducted using the EasyCycler 96 PCR system (Analytik Jena Corp., Germany). Subsequent to PCR, the amplification products from diverse samples were labeled with specific indices, pooled in equimolar ratios, and sequenced by Shanghai Mobio Biomedical Technology Co., Ltd. on the MiSeq platform (Illumina Inc., USA) following the stipulated manufacturer protocols.

### 2.4 Analysis of sequencing data

ASVs were identified with the DADA2 algorithm. The representative sequences for each ASV were annotated in reference to the SILVA database (SSU138). Alpha-diversity metrics, namely Shannon index for diversity, observed ASV count for richness, and the Pielou index for evenness, were calculated. Principal coordinates analysis (PCoA) based on Unweighted UniFrac distances was conducted by the R program (version 3.6.0, http://www.R-project.org/) to visualize microbiome space between samples. A heatmap plot of the key variables was generated using the ‘pheatmap’ package of the R program. For the identification of differentially abundant taxa, the linear discriminant analysis effect size (LEfSe) method (version 1.1, https://github.com/SegataLab/lefse) was deployed.

### 2.5 GC–MS analysis for targeted metabolomic of SCFAs

Upon thawing the serum on ice, 100 µL aliquots were transferred into a 2-mL glass centrifuge tube. This was combined with 100 μL of 20% phosphoric acid in water and 500 µL of 50 µg/mL 4-methyl valeric acid. After thorough vortex mixing, the suspension was centrifuged at 14,000×g for 20 minutes. A 1 µL aliquot of the resulting supernatant was analyzed using an Agilent 7890-5977 GC−MS system. For the quantification of short-chain fatty acids, a calibration curve was established spanning a concentration range of 0.1–100 µg/ml. The internal standard (IS) ensured corrections for potential inconsistencies in sample injection and minor instrument response variations. Chromatographic separation was performed on an Agilent FFAP capillary GC column (30 m × 0.25 mm ID × 0.25 µm). The initial column temperature was set at 100°C, then ramped to 160°C at a rate of 5°C/min. This was followed by an increase to 150°C at 5°C/min, and subsequently to 240°C at 80°C/min, where it was maintained for 6 minutes. Helium served as the carrier gas, flowing at 1.0 mL/min. The temperatures for the injection port and ion source were 260°C and 230°C, respectively. Analyses were conducted under SIM mode.

### 2.6 Isolation and culture of peripheral blood mononuclear cells

Ten-milliliter whole blood samples were collected from randomly selected GD patients (n=4) and healthy donors (n=4) into sterile tubes containing sodium heparin. The tubes were gently inverted to ensure thorough mixing of the blood. PBMCs were then isolated from the blood samples utilizing the Ficoll-Hypaque (GE Healthcare, USA) density gradient centrifugation method. Post-isolation, the PBMCs were resuspended in a cell culture medium consisting of RPMI 1640 supplemented with 10% heat-inactivated fetal bovine serum and 1% penicillin-streptomycin. The cells were seeded into 6-well plates at a density of 5 × 10^5^ cells per well. In the experimental setup, cells were either kept in the cell culture medium alone or subjected to various treatments: LPS (1 µg/mL), a combination of LPS and sodium acetate (2 mM), or LPS combined with a mixture of SCFAs [2 mM each of sodium acetate, sodium propionate, and sodium butyrate] (all reagents sourced from Sigma Aldrich, USA). After a 24-hour incubation at 37°C with 5% CO_2_, both the cells and their respective culture supernatants were harvested for subsequent analyses.

### 2.7 Flow cytometric analysis

For the assessment of B-cell subsets and CD32b expression, the collected whole blood samples from 33 GD patients and 32 HCs were subjected to a staining procedure with antibodies V500-CD45, APC-H7-CD19, BV421-IgD, PE-Cy7-CD27, and APC-CD32. After a 30-minute incubation at room temperature, erythrocytes were lysed using a standard lysing solution. For Th/Tc cell subset analysis, whole blood samples were treated with a cell activation cocktail, comprising phorbol-12-myristate-13-acetate, ionomycin, and brefeldin A, and incubated for 6 hours. Subsequent staining was conducted at room temperature for 30 minutes using the following antibodies: V500-CD45, FITC-CD45, APC-CD3, PE-CD3, and APC-H7-CD8. This was followed by cell fixation using a specialized fixative, permeabilization in a wash buffer, and staining with PerCp-Cy5.5-IFN-γ, APC-IL-4, BV421-IL-17, and PE-FoxP3 antibodies. Post-staining, cell samples were processed on a FACSCanto II flow cytometer. Data acquisition and analyses were conducted using the FACSDiva software (BD, USA). Comprehensive details regarding the reagents used can be found in the **Supplementary Methods** section.

### 2.8 Assay for LPS and inflammatory factors

Serum concentrations of LPS, LPS-binding protein (LBP), B-cell activating factor (BAFF), a proliferation-inducing ligand (APRIL), TNF-α, IL-17, IL-10, and IL-6 were accurately measured using human enzyme-linked immunosorbent assay kits provided by Cusabio Biotech (Cusabio, China) and Multi Science (Multi Science, China), following the manufacturer’s recommended protocols. Specifically, the assays used were CSB-E09945h for LPS, CSB-E09629h for LBP, CSB-E11912h for BAFF, CSB-E15012h for APRIL, CSB-E09315h for TNF-α, CSB-E12819h for IL-17, CSB-E04593h for IL-10, and 70-EK106/2-96 for IL-6.

Additionally, cytokine levels in supernatants from both stimulated and non-stimulated PBMC cultures were analyzed using a multi-analyte microsphere-based flow immunofluorescence assay kit (Ruisikeer Biotechnology, China). This process involved incubating each supernatant with antibody-conjugated microbeads for two hours at room temperature, followed by a 30-minute incubation with PE-conjugated secondary antibodies to form a sandwich complex. After washing the microbeads, the fluorescence intensity spectrum was assessed via FACSCanto II flow cytometry (BD, USA), in adherence to the manufacturer’s guidelines.

### 2.9 Statistical analysis

Data processing and statistical analyses were conducted using SPSS Statistics 20.0 (IBM, New York, USA). Continuous variables adhering to a normal distribution are expressed as mean ± standard deviation, while non-normally distributed data are presented as medians with interquartile ranges. For the comparison of two groups, Student’s *t*-test was applied to normally distributed continuous variables, and the Mann–Whitney *U* test was used for those not following a normal distribution. The Bonferroni test was employed to evaluate the differences among four stimulation groups. Categorical data were analyzed using the chi-square test. Correlational evaluations were performed using the Spearman rank correlation test. Statistical significance was set at a *P*-value < 0.05, with significance levels indicated as ^***^ *P* < 0.001, ^**^ *P* < 0.01, and ^*^ *P* < 0.05.

## 3 Results

### 3.1 Demographics and clinical characteristics of participants

**Table 1** provides a comparative overview of the clinical and demographic parameters between GD patients and HCs. There were no statistically significant differences in terms of sex, age, and body mass index (BMI) between the two groups. However, serum levels of FT3, FT4, TgAb, TPOAb, and TRAb were notably elevated in the GD group compared to the HCs, with the differences reaching statistical significance (*P* < 0.05).

**Table 1:** Demographics and clinical characteristics.

	Healhty controls (n = 45)	Graves’ disease patients (n = 52)	*P*
Sex (M/F)	5/40	9/43	0.386
Age (years)	33.0 (27.0-52.0)	37.0 (27.3-48.8)	0.769
BMI (kg/m^2^)	22.2 ± 2.6	21.5 ± 2.6	0.140
FT3 (pmol/L)	4.8 ± 0.6	27.7 ± 12.2	< 0.001
FT4 (pmol/L)	16.4 (14.6-17.8)	70.7 (46.5-100.0)	< 0.001
TSH (μIU/mL)	2.1 (1.7-2.8)	< 0.01	< 0.001
TgAb (IU/mL)	0.2 (0.2-0.2)	11.2 (2.3-110.6)	< 0.001
TPOAb (IU/mL)	0.7 (0.5-1.2)	220.0 (5.0-774.1)	< 0.001
TRAb (IU/L)	0.3 (0.3-0.8)	10.0 (5.8-19.3)	< 0.001

Data on BMI and FT3 were expressed as the means ± standard deviations. Data on age, FT4, TSH, TgAb, TPOAb and TRAb were expressed as the medians (25^th^–75^th^ percentile). BMI, body mass index; FT3, free triiodothyronine; FT4, free tetraiodothyronine; TSH, thyroid-stimulating hormone; TgAb, thyroglobulin antibody; TPOAb, anti-thyroid peroxidase antibody; TRAb, thyroid stimulating hormone receptor.

### 3.2 Distinct immune profiles characterize GD patients

**Figure 1:**
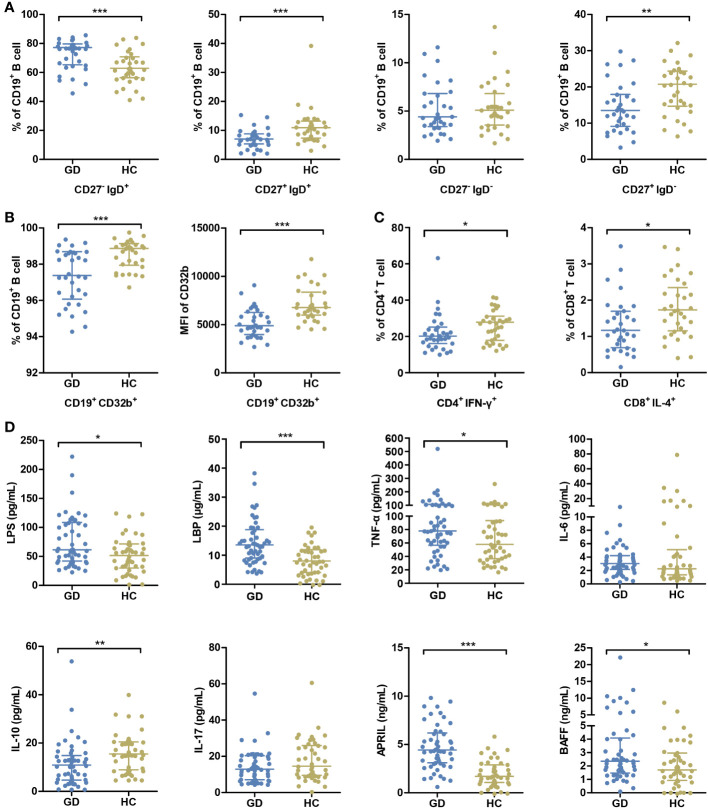
GD patients present abnormal immune status compared with HCs. **(A)** The percentages of peripheral blood B-cell subsets, including naive B cells (CD27^-^ IgD^+^), pre-switched memory B cells (CD27^+^ IgD^+^), double-negative memory B cells (CD27^-^ IgD^-^) and conventional memory B cells (CD27^+^ IgD^-^), were compared between GD patients and HCs. **(B)** The percentage of B cells expressing CD32b and the mean MFI values of CD32b on B cells were compared between GD patients and HCs. **(C)** The percentages of Th1 cells (CD4^+^ IFN-γ^+^) in CD4^+^ T cells and Tc2 cells (CD8^+^ IL-4^+^) in CD8^+^ T cells were compared GD patients and HCs. **(D)** The levels of serum LPS and inflammatory factors were compared between GD patients and HCs. GD, Graves’ disease; HCs, healthy controls; MFI, mean fluorescence intensity. * indicates P < 0.05, ** indicates P < 0.01, *** indicates P < 0.001.

A comprehensive assessment of the immune status was undertaken. While all participants underwent the cytokine analysis, flow cytometry assessment was completed for 33 GD patients and 32 HCs. In GD patients, there was a noticeable elevation in the percentage of naïve B cells compared to the HCs. Conversely, the percentages of conventional memory B cells, preswitched memory B cells, and Th1 and Tc2 cell subsets were diminished in the GD group (all *P* < 0.05, **Figures 1A, C**, **Supplementary Figure S1**, **S2**). Notably, the expression of CD32b, a pivotal immune checkpoint molecule, was markedly diminished in GD patients relative to HCs (*P* < 0.05, **Figure 1B**).

In parallel, relative to HCs, GD patients exhibited elevated serum concentrations of LPS, LBP, TNF-α, APRIL and BAFF. On the other hand, IL-10 levels were distinctly lower in GD patients (all *P* < 0.05, **Figure 1D**).

### 3.3 Distinct gut microbial and serum SCFA profiles in GD patients relative to HCs

**Figure 2:**
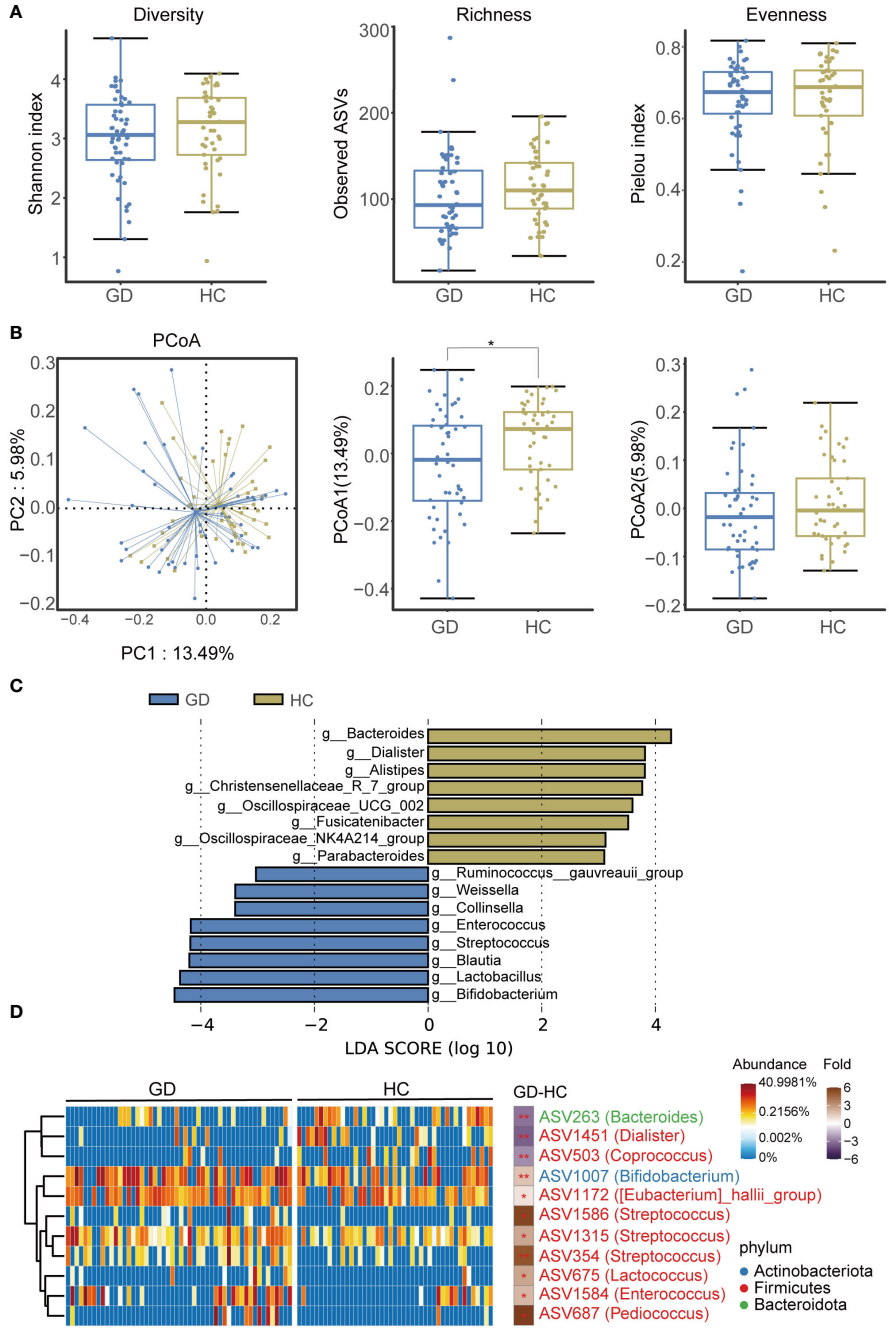
GD patients displayed alternation of gut microbiota compared with HCs. **(A)** Diversity, richness and evenness of gut microbiota were compared between GD patients and HCs. **(B)** The principal coordinates analysis (PCoA) showing the beta diversity between GD patients and HCs using PERMANOVA based on Bray-Curtis distances. **(C)** Linear discriminant analysis (LEfSe, LDA>3) column diagram. **(D)** Heatmap showing the relative abundance of the discriminatory ASVs that drive the differences between GD patients and HCs. GD, Graves’ disease; HCs, healthy controls. * indicates P < 0.05.

An assessment of ASV diversity—encompassing both richness and evenness—indicated no significant disparity between GD patients and HCs (**Figure 2A**, **Supplementary Figure S3**). To appraise the beta diversity of the gut microbiota, we employed the Unweighted UniFrac distance. PCoA discernibly differentiated the gut microbiota structure between the two groups (Adonis, R^2^ = 0.022, *P* < 0.05, **Figure 2B**).

We subsequently delved into the taxonomic architecture and variations of the gut microbiome. The bacterial community’s composition and relative abundance in each specimen at the phylum and genus levels are elucidated in **Supplementary Figure S4**. At the genus level, notable variations in relative abundance between the two groups were observed for genera such as *Blautia*, *Bifidobacterium*, *Bacteroides*, *Lactobacillus*, *Streptococcus*, *Enterococcus*, *Dialister*, and *Pediococcus* (**Supplementary Figure S5**). LEfSe analysis highlighted an augmented representation of *Blautia*, *Bifidobacterium*, *Lactobacillus*, *Streptococcus*, and *Enterococcus* in the GD patients relative to HCs, while genera like *Bacteroides*, *Dialister*, and *Alistipes* were subdued (**Figure 2C**).

Further, a total of 11 divergent ASVs were identified in the GD group compared to HCs. These included ASV263(*Bacteroides*), ASV1451(*Dialister*), ASV503(*Coprococcus*), ASV1007(*Bifidobacterium*), ASV1172(*[Eubacterium]_hallii_group*), ASV1586(*Streptococcus*), ASV1315(*Streptococcus*), ASV354(*Streptococcus*), ASV675(*Lactobacillus*), ASV1584(*Enterococcus*), and ASV687(*Pediococcus*) (**Figure 2D**).

**Figure 3:**
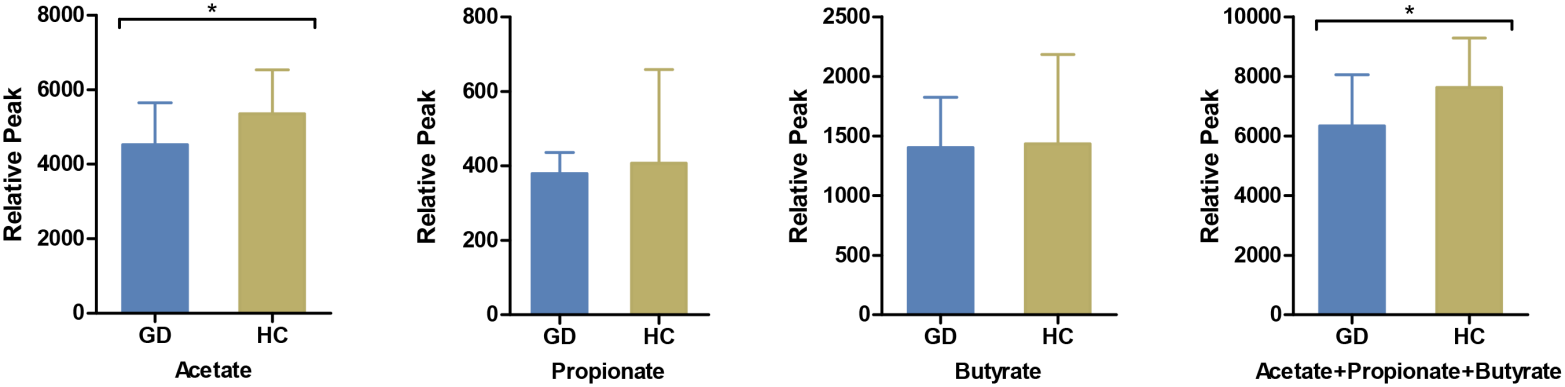
Comparison of serum short-chain fatty acids between GD patients and HCs. The levels of serum acetate, propionate, butyrate and their cumulative mix were compared between GD patients and HCs. GD, Graves’ disease; HCs, healthy controls. * indicates P < 0.05.

Gut metabolites, with a specific emphasis on SCFAs, play a pivotal role in facilitating interactions between intestinal flora and the host immune system. In this context, we quantified salient immunomodulatory SCFAs, including acetate, propionate, and butyrate. Notably, acetate levels were markedly reduced in GD patients compared to HCs (**Figure 3**).

### 3.4 Association of gut microbiota dysbiosis and metabolites with immune markers and clinical indices

**Figure 4:**
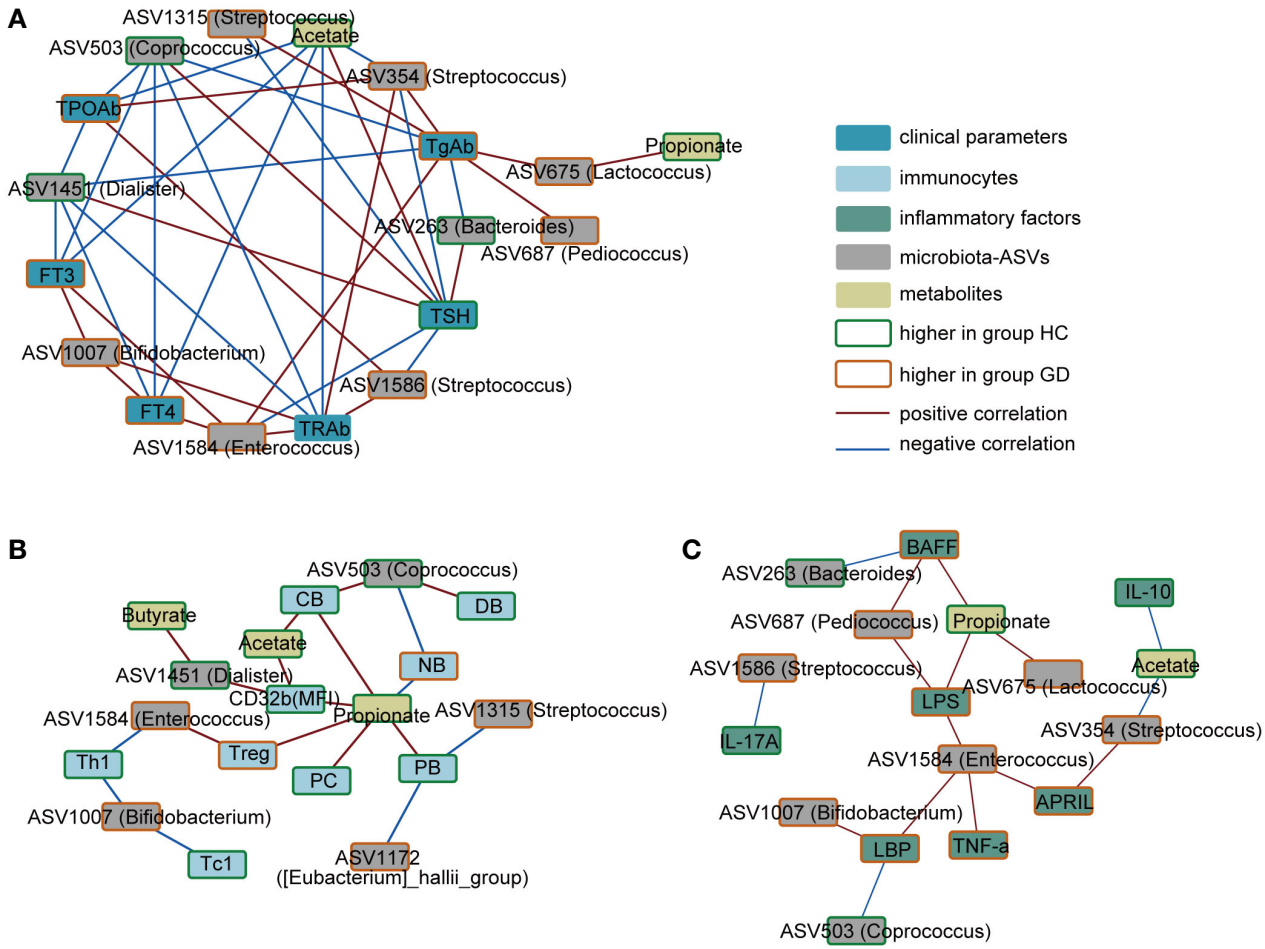
Correlation network of key ASVs, metabolites, immune indicators and clinical phenotypes. **(A)** Correlation in the key ASVs, metabolites and clinical phenotypes. **(B)** Correlation in the key ASVs, metabolites and immunocytes. **(C)** Correlation in the key ASVs, metabolites and inflammatory factors. Red connections indicate the positive correlation (FDR < 0.05), whereas blue connections show correlations that were negative (FDR < 0.05). ASV, amplicon sequence variant; FT3, free triiodothyronine; FT4, free tetraiodothyronine; TSH, thyroid-stimulating hormone; TgAb, thyroglobulin antibody; TPOAb, anti-thyroid peroxidase antibody; TRAb, thyroid stimulating hormone receptor; NB,native B cells; PB, preswitched memory B cells; DB, double-negative memory B cells; CB, conventional memory B cells; PC, plasma cells; LPS, lipopolysaccharide; LBP, LPS-binding protein; BAFF, B-cell-activating factor; APRIL, a proliferation-inducing ligand.

To elucidate the possible links between specific bacterial taxa, metabolites, and immune disturbances observed in GD, a correlation analysis was performed. As depicted in **Figure 4**, serum TRAb levels exhibited a positive association with ASV354(*Streptococcus*), ASV1586(*Streptococcus*), ASV1007(*Bifidobacterium*), and ASV1584(*Enterococcus*). In contrast, negative correlations were observed with ASV1451(*Dialister*), ASV503(*Coprococcus*), and acetate. Notably, acetate levels inversely correlated with ASV354(*Streptococcus*) but showed a positive association with conventional memory B cells and the expression of CD32b. Furthermore, ASV1584(*Enterococcus*) demonstrated a positive correlation with LPS and pro-inflammatory markers, including TNF-α, APRIL and LBP.

### 3.5 *In vitro* modulation of cytokines by LPS and SCFAs

**Figure 5:**
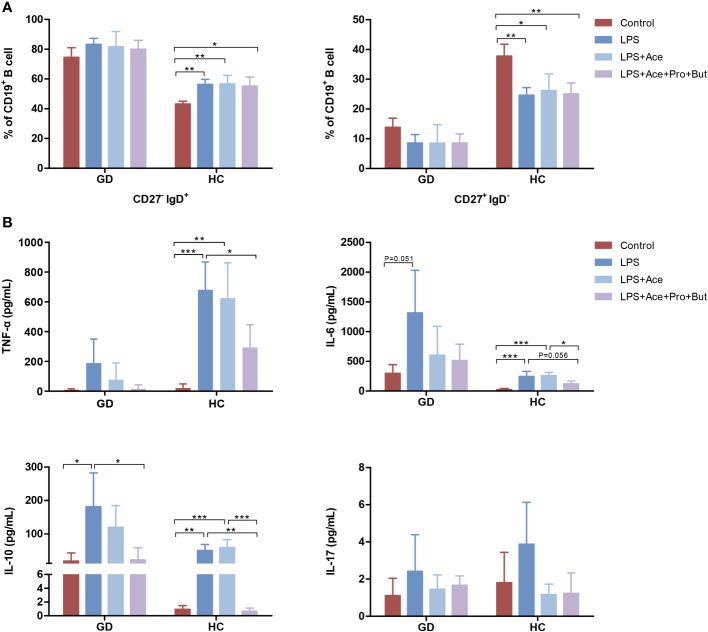
Effects of LPS and short-chain fatty acids on B-cell subsets distribution and cytokines production in GD patients and HCs. **(A)** Quantifies the distribution of B-cell subsets in PBMC cultures stimulated with LPS alone, LPS combined with Ace, LPS with Ace, Pro, and But, compared to unstimulated PBMC cultures. **(B)** Evaluates the production of inflammatory cytokines under the same conditions. LPS, lipopolysaccharide; Ace, acetate; Pro, propionate; But, butyrate; PBMC, peripheral blood mononuclear cell. GD, Graves’ disease; HCs, healthy controls. * indicates P < 0.05, ** indicates P < 0.01, *** indicates P < 0.001.

To further decipher the implications of LPS and SCFAs in immune aberrations observed in GD, PBMCs were treated with both LPS and SCFAs. LPS stimulation in healthy individuals resulted in a notable increase in the the percentage of naive B cells, accompanied by a reduction in the conventional memory B-cell population. Nevertheless, this LPS-induced shift in B-cell subsets was not mitigated by SCFAs (**Figure 5A**). Conversely, such changes were absent in individuals with GD.

Moreover, LPS exposure markedly augmented the production of TNF-α, IL-6, and IL-10. This enhancement was notably suppressed when acetate was combined with propionate and butyrate. However, acetate on its own did not yield this inhibitory effect (**Figure 5B**). Interestingly, the extent of cytokine response differed between GD patients and HCs upon LPS exposure. GD patients exhibited a higher propensity for IL-6 production, whereas HCs showed a greater increase in TNF-α levels (**Figure 5B**), highlighting the differential immune response dynamics in GD compared to healthy states.

## 4 Discussion

In prior research, fecal microbiota transplantation from GD patients was shown to notably elevate the incidence of GD in mice and increase serum thyroid hormone and proinflammatory cytokine levels (Su et al., 2020). This underscores the pivotal role of gut microbiota aberrations in GD’s pathogenesis, suggesting they act as more than mere secondary effects or concomitant phenomena. As delineated previously, autoimmunity primarily drives GD pathogenesis. Yet, the exact factors instigating the immune imbalance in GD remain elusive. It is thus imperative to probe whether gut dysbiosis is central to GD’s autoimmune pathogenesis.

In this study, we adopted the ASVs methodology, in lieu of OTU, as our clustering algorithm to gauge bacterial relative abundances. Our findings revealed a marked alteration in the microbial community structure of GD patients compared to HCs. Echoing previous research (Yan et al., 2020; Yang et al., 2022), we discerned elevated abundances of ASV675(*Lactobacillus*) and ASV1007(*Bifidobacterium*) in GD, with ASV1007(*Bifidobacterium*) manifesting positive correlations with FT3, FT4, and TRAb levels. An animal-based study similarly identified a positive association between *Lactobacillus* and FT4 (Masetti et al., 2018). Such observations insinuate a potential pathogenic influence of these two genera in GD. Furthermore, specific strains of *Lactobacillus* and *Bifidobacterium* possess amino acid sequences mirroring those of TPO and Tg (Kiseleva et al., 2011). Consequently, the augmented prevalence of these potentially pathogenic strains could initiate autoimmune reactions via molecular mimicry mechanisms.

Additionally, a primary cluster of bacterial genera—including ASV1584(*Enterococcus*), ASV1586(*Streptococcus*), ASV1315(*Streptococcus*), and ASV354(*Streptococcus*)—exhibited enrichment in GD patients. Notably, these ASVs exhibited correlations with thyroid hormones, thyroid autoantibodies, cytokines, and lymphocyte subsets, hinting at their possible immunomodulatory properties. Elevated *Enterococcus* prevalence has been documented in rheumatoid arthritis and systemic lupus erythematosus studies (Kim et al., 2019; Mena-Vázquez et al., 2020). Particularly, *Enterococcus gallinarum* has been shown to transgress the gut barrier, colonizing internal organs and stimulating autoimmunity in hosts (Fine et al., 2020), pointing towards the potential pathogenic role of ASV1584(*Enterococcus*) in GD. *Streptococcus* has been implicated in compromising epithelial integrity and inciting inflammatory responses (Cui et al., 2020). Though its role in GD’s pathogenesis remains unelucidated, heightened *Streptococcus* levels were associated with APRIL over-expression. Conversely, abundances of ASV263(*Bacteroides*) and ASV1451(*Dialister*) witnessed a notable decline in GD. Previous research noted diminished *Bacteroides* spp. in European GD patients (Biscarini et al., 2023), while an opposing trend was observed in GD patients from eastern China (Jiang et al., 2021). Such discrepancies can be attributed mainly to regional variations and clustering algorithm methodologies. Nonetheless, these genera are universally acknowledged as SCFA-producing microbes, and their decreased prevalence is observable in disorders like depression and atherosclerosis (Yoshida et al., 2018; Yu et al., 2023). Moreover, these genera exhibited negative correlations with thyroid autoantibodies. In summary, it is compelling to delve into whether microbial production and metabolites dictate the immune profile of GD.

In our study, we observed a significant elevation of LPS, an endotoxin derived from gram-negative bacteria, in GD patients. Prior studies have indicated that LPS can trigger proliferation and differentiation of murine B-cells (Lu and Munford, 2016). Furthermore, following the intravenous administration of LPS, memory B cells experienced partial depletion from the circulatory system in healthy male subjects (Brinkhoff et al., 2019). Our *in vitro* findings align with these *in vivo* observations, showing a reduction in conventional memory B-cell subsets in healthy individuals following LPS exposure, mirroring the immune status seen in GD. This alteration can be partially ascribed to Fas-induced apoptosis, steered by the LPS/TLR4 signaling pathway (Chang et al., 2016). Collectively, these insights underscore LPS’s pivotal role in the skewed distribution of B-cell subsets.

Moreover, we evaluated the expression of CD32b, a key inhibitory receptor, to ascertain the B cells’ immune status within our study group. There was a noticeable reduction in CD32b expression in GD patients. Interestingly, in our *in vitro* experiments, LPS stimulation did not markedly alter CD32b expression (data not presented). This indicates that the diminished expression of inhibitory receptors in GD might operate independently of LPS. Complementing this, Swanson-Mungerson et al. (Swanson-Mungerson et al., 2017) highlighted that LPS prompts B-cell activation by enhancing the expression of MHCII and CD86. In summary, LPS seems to instigate B-cell activation primarily by augmenting the expression of co-stimulatory molecules, rather than by suppressing inhibitory receptor expression.

Numerous investigations have documented anomalous cytokine production in GD patients. In line with these findings, our data indicated elevated levels of TNF-α in GD patients relative to HCs, while there was a decrease in IL-10 expression. Even though IL-10 is typically regarded as an anti-inflammatory molecule, we observed its upregulated production *in vitro* upon LPS stimulation. This is congruent with prior research (Schildberger et al., 2013). The pronounced induction of IL-10 might suggest an initiation of counteractive, anti-inflammatory reactions. Thus, the compromised serum IL-10 in GD patients may contribute to the observed immune dysregulation. Pivotal inflammatory mediators, including IL-17, TNF-α, and IL-6, are integral to GD pathophysiology. IL-17, a notable proinflammatory cytokine, instigates the NF-κB signaling cascade, resulting in the subsequent release of TNF-α and IL-6 (Lu et al., 2023). Concurrently, heightened activity of the TNF-α/TNFR or IL-6/STAT3 pathways also promotes Th17 differentiation and IL-17 production (Pesce et al., 2022). This culminates in a self-amplifying loop, further intensifying immune-mediated damage to the thyroid. Consequently, mitigating these pro-inflammatory cytokines emerges as a pivotal strategy in averting GD progression. Previous studies have shown that acetate reduces IL-17 production and the proportion of Th17 cells (Wibowo et al., 2021). In our experiments, the increase in IL-17 production following LPS exposure was not significant, casting uncertainty on the protective role of SCFAs. As for TNF-α and IL-6, the inhibitory impact of sodium acetate on their release was not marked. However, both propionate and butyrate have demonstrated potential in curtailing their synthesis (Segain et al., 2000; Lang et al., 2022). Remarkably, we discerned a substantial decline in the release of these proinflammatory cytokines when exposed to a combination of sodium acetate, propionate, and butyrate. Such multifaceted findings suggest that acetate collaborates synergistically with other SCFA components in countering inflammation.

The present study is not without limitations. Firstly, while we employed 16S rRNA analysis to delineate the microbial composition, this approach did not enable us to discern at the species level. Future investigations leveraging metagenomic techniques could further refine our understanding and pinpoint specific pathogens associated with GD. Secondly, our immune profiling was based on samples derived from peripheral blood. It is noteworthy that circulating lymphocytes might exhibit differences from immune cells procured from the thyroid or lymphatic tissues.

Our research uncovered a correlation between gut dysbiosis and various aspects of thyroid health, including thyroid function, autoantibodies, and overall immune status. Furthermore, gut dysbiosis appeared to influence the distribution of B-cell subsets and cytokine production, indicating a potential role in the autoimmunity associated with GD. These insights contribute to a growing body of evidence on the involvement of gut microbiota in GD, suggesting that further exploration into its role could significantly deepen our understanding of GD’s pathogenesis.

## Data availability statement

The datasets presented in this study can be found in online repositories: NCBI database now (https://www.ncbi.nlm.nih.gov/sra/?term=PRJNA799831).

## Ethics statement

The studies involving humans were approved by Ethics Committee of Henan Provincial People’s Hospital. The studies were conducted in accordance with the local legislation and institutional requirements. The participants provided their written informed consent to participate in this study. The manuscript presents research on animals that do not require ethical approval for their study.

## Author contributions

YL: Conceptualization, Funding acquisition, Writing – original draft. ST: Conceptualization, Writing – original draft. YF: Data curation, Resources, Writing – review & editing. BX: Project administration, Validation, Writing – review & editing. CC: Project administration, Validation, Writing – review & editing. YS: Data curation, Resources, Writing – review & editing. WW: Data curation, Resources, Writing – review & editing. LZ: Data curation, Writing – review & editing. ZH: Investigation, Writing – review & editing. XS: Data curation, Supervision, Writing – review & editing. YYF: Validation, Writing – review & editing. JY: Supervision, Validation, Writing – review & editing. YZ: Funding acquisition, Writing – review & editing. XD: Visualization, Writing – review & editing. LW: Software, Writing – review & editing. HR: Visualization, Writing – review & editing. CW: Methodology, Writing – review & editing. HY: Conceptualization, Funding acquisition, Writing – review & editing.
