# Supplementary material for: Alteration in gut microbiota is associated with immune imbalance in Graves’ disease
Source: Front Cell Infect Microbiol. 2024 Mar 12;14:1349397. doi: 10.3389/fcimb.2024.1349397 (PMC10963416; doi:10.3389/fcimb.2024.1349397)
Supplement: Supplementary file 1 [file DataSheet_1.docx]

Supplementary Material

# Supplementary Methods

## Inclusion and exclusion criteria of participants

All subjects were of Han nationality and were born in central plains of China. GD patients were diagnosed based on typical clinical presentation, the presence of hyperthyroidism, positive serum TRAb or high radioactive iodine uptake. All 52 GD patients were newly diagnosed or relapsed patients without any treatment. In addition, the control group consisted of 45 healthy volunteers who visited the hospital for their annual physical examination. The HCs had no medical or family history of thyroid diseases and had normal thyroid function and negative autoantibody responses.

The following exclusion criteria were applied to all groups: (1) body mass index (BMI) ≥ 28Kg/m^2^; (2) had a continuous antibiotic use history for >3 days within 3 months prior to enrolment; (3) had diabetes mellitus, malignancy or infectious diseases; (4) had other autoimmune disease, such as SLE, RA, IBD, T1DM and multiple sclerosis (MS); (5) had immunosuppressive drug; (6) had undergone gastrectomy, fundoplication, colostomy or other digestive system surgery; (7) had persistent vomiting or a suspected gastrointestinal obstruction; (8) had severe cardiovascular and cerebrovascular diseases or liver, kidney and haematopoietic system diseases; (9) had alcoholism (drinking more than 5 times in one week, more than 100 g of spirits, 250 g of rice wine or 5 bottles of beer); (10) were pregnant.

# Supplementary Table and Figures

## Supplementary Table

## Supplementary Table S1. Detail information of reagents

| Reagent | Corporation | Catalog No. | Country |
| --- | --- | --- | --- |
| V500-CD45 | BD Biosciences | 560777 | USA |
| APC-H7-CD19 | BD Biosciences | 560177 | USA |
| BV421-IgD | BD Biosciences | 562518 | USA |
| PE-Cy7-CD27 | BD Biosciences | 560609 | USA |
| Percp-Cy5.5-CD138 | BD Biosciences | 564605 | USA |
| APC-CD32 | BD Biosciences | 559769 | USA |
| APC-H7-CD8 | BD Biosciences | 560179 | USA |
| PerCp-Cy5.5-IFN-γ | BD Biosciences | 560742 | USA |
| APC-IL-4 | BD Biosciences | 560671 | USA |
| BV421-IL-17 | BD Biosciences | 562933 | USA |
| PE-Cy7-CD25 | BD Biosciences | 557741 | USA |
| PE-FoxP3 | BD Biosciences | 560852 | USA |
| PE-CD3 | BioLegend | 300308 | USA |
| APC-CD3 | BioLegend | 300312 | USA |
| FITC-CD45 | BioLegend | 304006 | USA |
| Lysing buffer | BD Biosciences | 555899 | USA |
| Leukocyte Activation Cocktail | BD Biosciences | 550583 | USA |
| Foxp3 / Transcription Factor Staining Buffer Set | eBioscience | 00-5523-00 | USA |

## Supplementary Figures

**
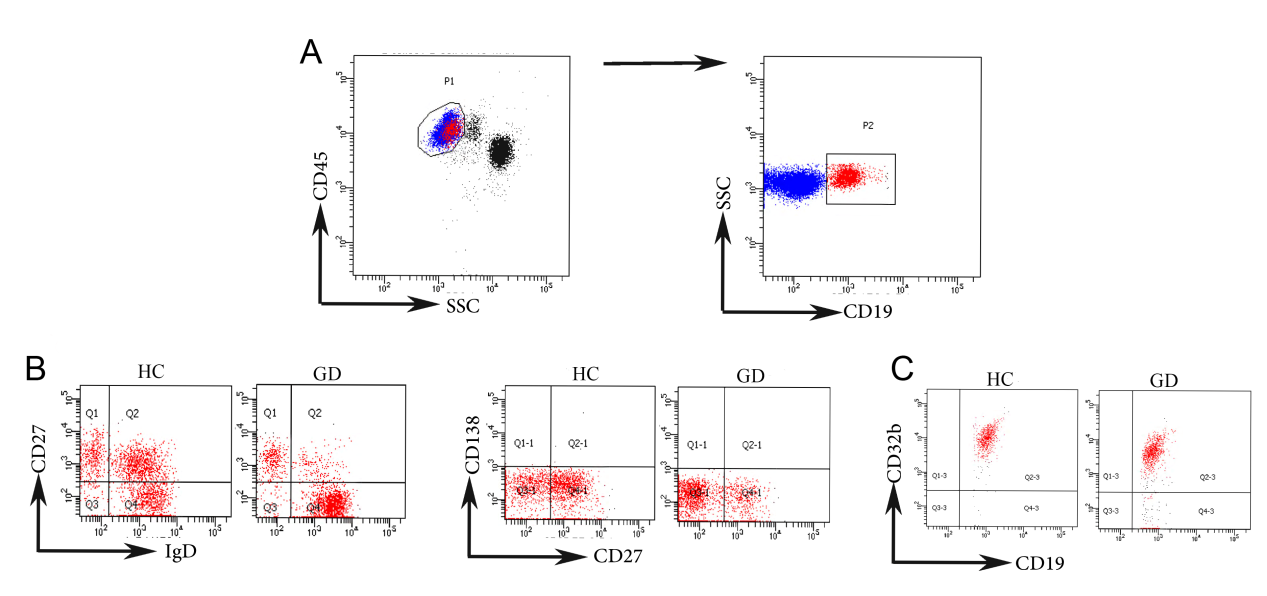
**

**Supplementary Figure S1.** Flow cytometry data of B cell subsets and inhibitory receptor CD32b expression. (A) Gating strategy. The lymphocytes (P1) were firstly gated on a SSC/CD45 dot plot. On these cells, a CD19/SSC dot plot allowed the definition of CD19^+^ B cells (P2). (B) Representative flow cytometry dot plots example of B cell subsets in GD patients and HCs. From the gate 2 (P2), B cells were subdivided into naïve B cells (CD27^-^IgD^+^), preswitched memory B cells (CD27^+^IgD^+^), conventional memory B cells (CD27^+^IgD^-^), double-negative memory B cells (DN, CD27^-^IgD^-^) and plasma cells (PCs, CD27^+^CD138^+^) subsets. (C) Representative flow cytometry dot plots example of inhibitory receptor CD32b expression on B cells in GD patients and HCs. From gate 2 (P2), CD32b expression on B cells was shown on a CD19/CD32b dot plot. GD, Graves' disease; HCs, healthy controls.

**
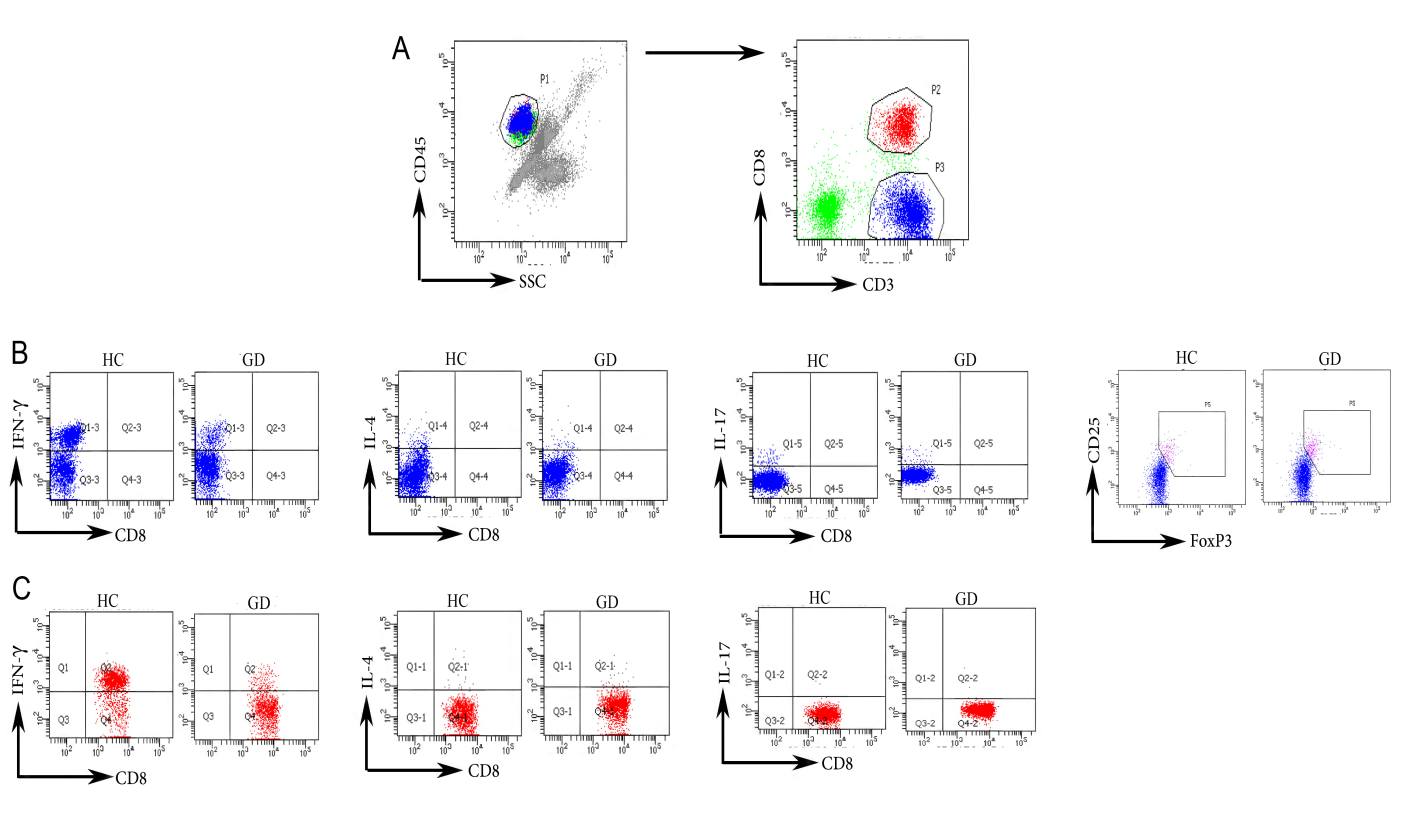
**

**Supplementary Figure S2.** Flow cytometry data of Th/Tc subsets. (A) Gating strategy. The lymphocytes (P1) were firstly gated on a SSC/CD45 dot plot. On these cells, a CD3/CD8 dot plot allowed the definition of CD4^+^ T cells (CD8^-^ T cells, P3) and CD8^+^ T cells (P2). (B) Representative flow cytometry dot plots example of Th subsets in GD patients and HCs. From the gate 3 (P3), CD4^+^ T cells were subdivided into Th1 (CD4^+^IFN-γ^+^), Th2 (CD4^+^IL-4^+^), Th17 (CD4^+^IL-17^+^) and Treg (CD4^+^CD25^+^FoxP3^+^) subsets. (C) Representative flow cytometry dot plots example of Tc subsets in GD patients and HCs. From the gate 2 (P2), CD8^+^ T cells were subdivided into Tc1 (CD8^+^IFN-γ^+^), Tc2 (CD8^+^IL-4^+^) and Tc17 (CD8^+^IL-17^+^) subsets. GD, Graves' disease; HCs, healthy controls.

**
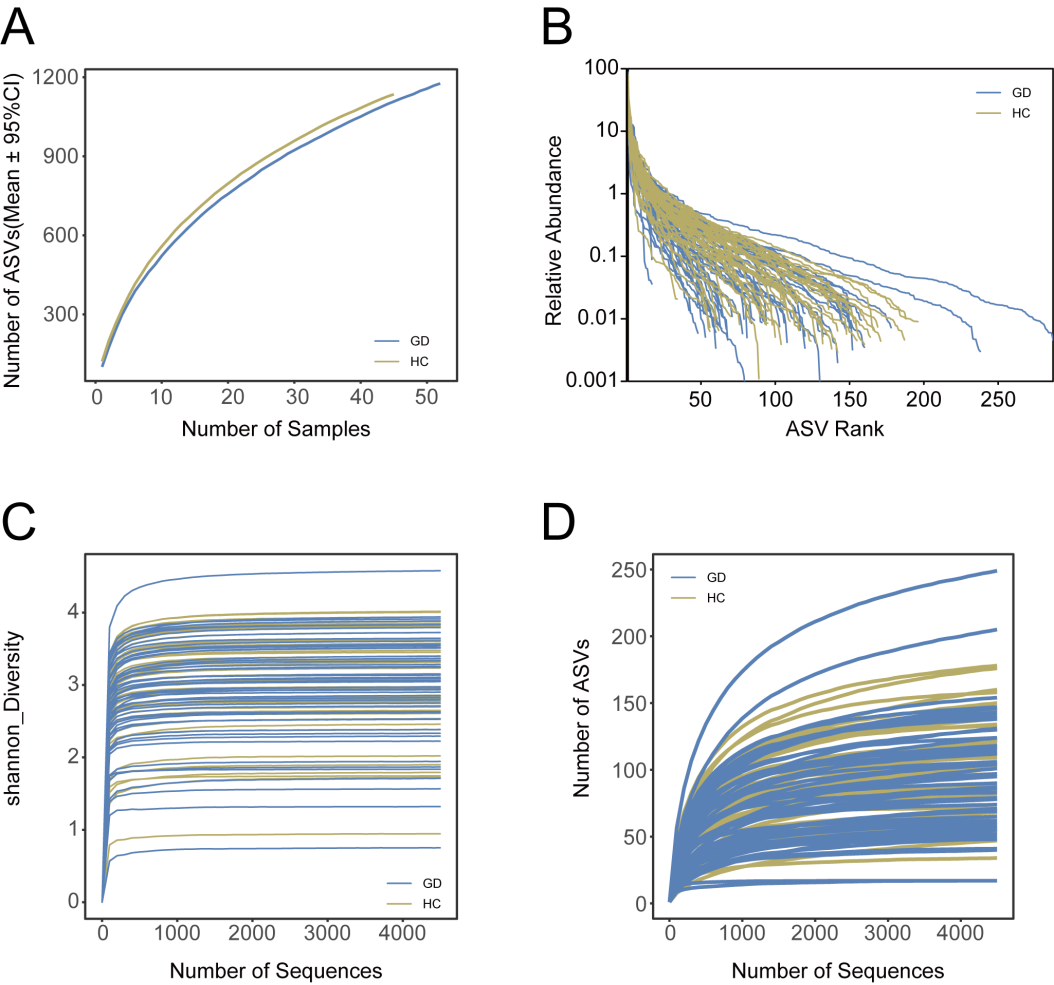
**

**Supplementary Figure S3.** The detailed values of gut microbial diversity index between GD patients and HCs. (A) The rarefaction analysis between the number of samples and the number of ASVs. As the number of samples increased, the number of ASVs approached saturation in GD patients and HCs. (B) A rank-abundance distribution curve for the ASVs of GD patients and HCs. (C) A shannon-wiener curve between the number of sequences in GD patients and HCs. (d) A rarefaction curve between the number of ASVs and the number of sequences in GD patients and HCs. GD, Graves' disease; HCs, healthy controls; ASV, amplicon sequence variant.


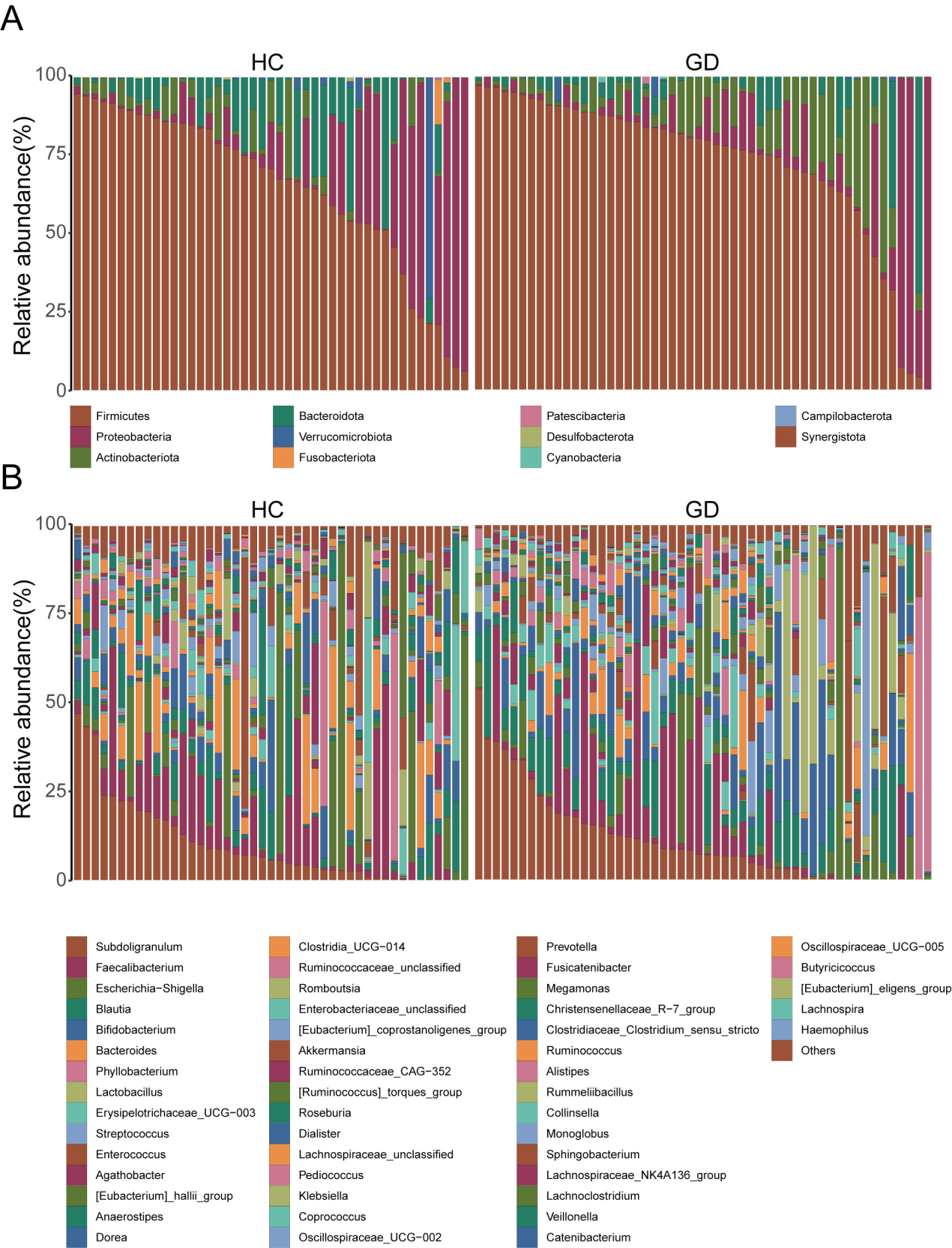


**Supplementary Figure S4.** The composition of bacterial community in GD patients and HCs. The composition and abundance of bacterial community at the phylum (A) and genus (B) level in each sample of the participants. GD, Graves' disease; HCs, healthy controls.


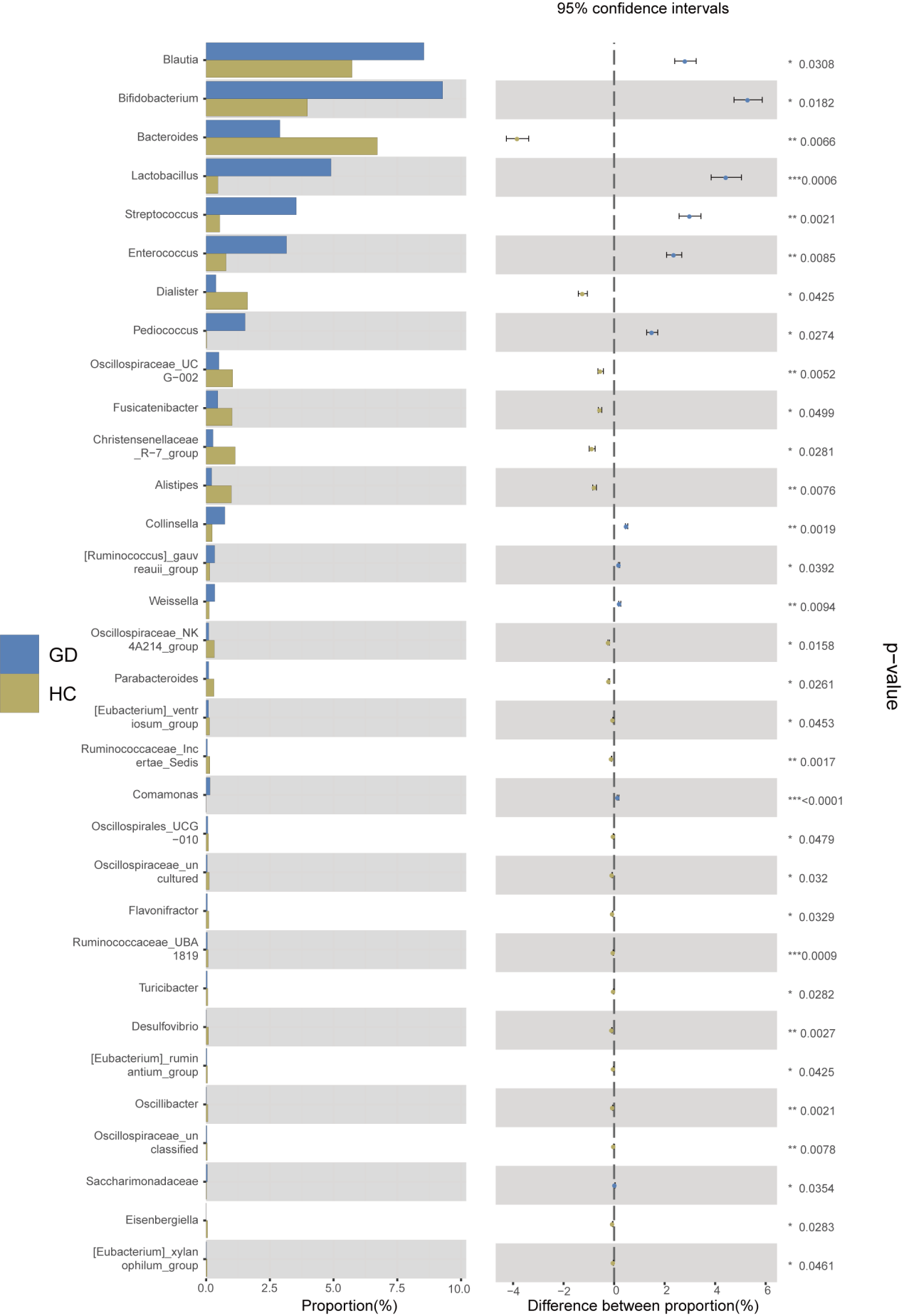


**Supplementary Figure S5.** Abundance of gut microbiota between GD patients and HCs. The relative abundances of the bacterial community were compared between GD patients and HCs at the genus levels. GD, Graves' disease; HCs, healthy controls.
